# Longitudinal changes in superficial microvasculature in glaucomatous retinal nerve fiber layer defects after disc hemorrhage

**DOI:** 10.1038/s41598-020-79151-y

**Published:** 2020-12-16

**Authors:** Yoko Okamoto, Tadamichi Akagi, Kenji Suda, Takanori Kameda, Masahiro Miyake, Hanako Ohashi Ikeda, Eri Nakano, Akihito Uji, Akitaka Tsujikawa

**Affiliations:** Department of Ophthalmology and Visual Sciences, Kyoto University Graduate School of Medicine, 54 Kawahara-cho, Shogoin, Sakyo-ku, Kyoto, 606-8507 Japan

**Keywords:** Optic nerve diseases, Retinal diseases

## Abstract

Glaucoma is a multifactorial optic neuropathy, possibly involving vascular dysfunction, leading to the death of retinal ganglion cells and their axons. Disc hemorrhage (DH) is known to be closely associated with the widening of retinal nerve fiber layer defect (NFLD); however, it has not been well elucidated how DH affects retinal microvasculature. We aimed to investigate the association between DH history and longitudinal changes in superficial retinal microvasculature in NFLD. We enrolled 15 glaucoma patients with DH history (32 glaucomatous NFLD locations, with or without DH history). NFLD-angle, superficial retinal vessel density (VD), and decreased superficial retinal microvasculature (deMv)-angle were assessed using optical coherence tomography angiography for at least three times over time. The mean follow-up period and OCT/OCTA scan interval were 21.3 ± 5.4 months (range, 12–28) and 6.8 ± 0.4 months (range, 2–18), respectively. Linear mixed-effects models showed that the presence of DH history was significantly associated with an additional NFLD-angle widening of 2.19 degree/year (*P* = 0.030), VD decrease of 1.88%/year (*P* = 0.015), and deMv-angle widening of 3.78 degree/year (*P* < 0.001). These changes were significantly correlated with each other (*P* < 0.001). Thus, the widening of NFLD was closely associated with deMv, and DH was associated with a subsequent decrease in superficial retinal microvasculature in NFLD.

## Introduction

Glaucoma is a progressive optic neuropathy characterized by the degeneration of retinal ganglion cells and their axons and results in visual field loss^1^. Widening of the retinal nerve fiber layer (RNFL) defect (NFLD) is an important sign of glaucoma progression, leading to the functional deterioration of the visual field^2,3^.

Recently, optical coherence tomography angiography (OCTA) has enabled noninvasive assessments of retinal microvasculature, and several studies using OCTA have revealed decreased superficial retinal vessel density (VD) in eyes with glaucoma^4–7^. It was also reported that a region of decreased superficial retinal microvasculature (deMv) in the parapapillary region was topographically associated with NFLD^6–8^.

Disc hemorrhage (DH) is well-known as an important risk factor for the progression of glaucoma, including visual field defects^9–12^ and RNFL thinning^13,14^. Recently, some studies have reported close associations between DH and parapapillary choroidal microvasculature dropout (CMvD) assessed using OCTA^15,16^. However, it has not been well elucidated how DH affects longitudinal change in retinal microvasculature.

In the present study, we investigated the longitudinal changes in NFLD, parapapillary superficial retinal VD, and parapapillary deMv in patients with glaucomatous NFLD with or without DH history and the association among these structural or vascular parameters.

## Results

### Demographic and clinical characteristics

**Figure 1:**
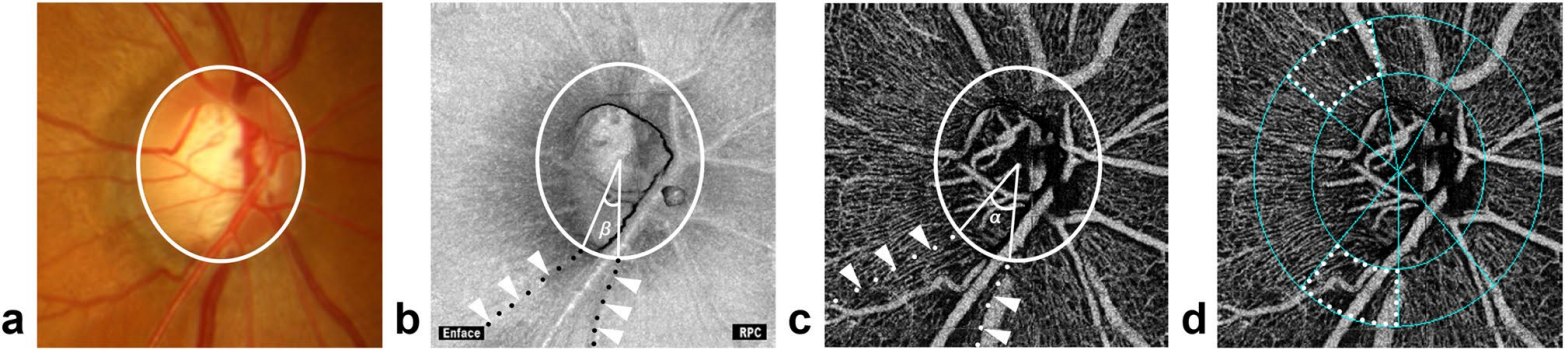
Measurements of NFLD-angle, VD, and deMv-angle. (**a**) Disc photograph. The white circle indicates the boundary of the optic disc. (**b**) NFLD-angle (β) is determined in the OCT en face image. RPC = Radial peripapillary capillary. (**c**) deMv-angle (α) is determined in the superficial OCTA image. (**d**) VD is assessed in the superficial OCTA images.

Fifteen glaucoma patients with DH history were enrolled, and a total of 32 NFLDs of 26 eyes were included in the analysis (Table 1). DH was detected within 3 years before the first OCTA examination in 18 NFLDs (DH group) and not in 14 NFLDs (non-DH group). NFLD-angle, VD, and deMv-angle were assessed in each NFLD quadrant, as shown in Fig. 1.

**Table 1:** Demographic and clinical characteristics of included subjects (N = 26 eyes of 15 subjects).

**By subjects (N = 15)**	
Age (years)	53.7 ± 9.1 (38–73)
Sex (F/M), n	8/7
**By eye (N = 26)**	
Diagnosis (POAG/PPG), n	24/2
Intraocular pressure (mmHg)	13.9 ± 2.7 (10–21)
Axial length (mm)	25.1 ± 1.2 (22.9–27.0)
Central corneal thickness (μm)	525.0 ± 36.8 (436–583)
Visual field mean deviation (dB)	− 3.71 ± 3.34 (− 13.07 to − 0.22)
Medication-baseline	1.4 ± 1.3 (0–4)
Medication-at last	2.0 ± 1.1 (0–4)
OCTA follow-up period (month)	21.3 ± 5.4 (12–28)

Data (except sex and diagnosis) are presented as mean ± standard deviation with the minimum and maximum values in parentheses.

*POAG* primary open angle glaucoma, *PPG* preperimetric glaucoma, *OCTA* optical coherence tomography angiography.

### Comparisons between DH and non-DH groups

Table 2 shows a comparison between DH and non-DH groups. There were 7 superotemporal NFLDs without DH, 7 inferotemporal NFLDs without DH, 6 superotemporal NFLDs with DH, and 12 inferotemporal NFLDs with DH. The means ± standard deviations of follow-up period and OCT/OCTA scan interval were 21.3 ± 5.4 months (range, 12–28) and 6.8 ± 0.4 months (range, 2–18), respectively. There were no significant differences in age, intraocular pressure (IOP), axial length, central corneal thickness, the number of glaucoma medications, follow-up period, and the number of OCT and OCTA examinations between groups (all *P* > 0.05). The mean NFLD-angle, VD, and deMv-angle at baseline were not significantly different between the groups (all *P* > 0.05) (Table 2). The NFLD-angle, VD, and deMv-angle at baseline were not significantly different between the superotemporal and inferotemporal quadrants (all *P* > 0.05) (Supplementary Table 1). In the NFLDs with DH, the differences between the baseline and final measurements of NFLD-angle and deMv-angle were statistically significant (NFLD-angle, 4.71 ± 5.97 degree, *P* < 0.001; VD, − 1.21 ± 3.49%, *P* = 0.16; deMv-angle, 10.24 ± 8.66 degree, *P* < 0.001), whereas, in the NFLDs without DH, the differences in measurements of NFLD-angle, VD, and deMv-angle were not significant (all, *P* > 0.05). The interobserver reproducibility of measurements of NFLD-angle and deMv-angle were intraclass correlation coefficient (ICC)(2,1) = 0.978 and ICC(2,1) = 0.955, respectively, which were excellent.

**Table 2:** Comparison of clinical characteristics between NFLD with DH and NFLD without DH (N = 32 NFLD locations).

	NFLD without DH (N = 14)		NFLD with DH (N = 18)		*P* value*
	Mean ± SD	(Range)	Mean ± SD	(Range)	
Age-baseline (years)	55.1 ± 9.1	(38–73)	52.8 ± 9.8	(38–73)	0.18
Mean IOP (mmHg)	13.9 ± 2.9	(9.9–20.3)	13.7 ± 2.6	(9.9–19.0)	0.99
Axial length (mm)	25.1 ± 1.3	(22.9–27.0)	24.9 ± 1.2	(22.9–27.0)	0.48
Central corneal thickness (μm)	523.0 ± 39.6	(436–583)	523.0 ± 39.6	(467–581)	0.77
Visual field mean deviation (dB)	− 4.4 ± 3.8	(− 13.1 to − 0.22)	− 3.2 ± 1.9	(− 7.89 to 0.35)	0.31
Medication-baseline	1.5 ± 1.5	(0–4)	1.6 ± 1.2	(0–4)	0.72
Medication-at last	1.9 ± 1.3	(0–4)	2.6 ± 1.0	(1–4)	0.48
OCTA follow-up period (month)	20.6 ± 5.6	(12–25)	22.6 ± 5.3	(12–28)	> 0.99
The frequency of OCTA exam	4.3 ± 1.2	(3–6)	4.2 ± 1.1	(3–6)	> 0.99
CMvD (+ /−), no	4/10		10/8		0.14
NFLD location (superotemporal/inferotemporal), no	7/7		6/12		0.36
deMv-angle-baseline (°)	32.9 ± 12.6	(12.7–54.6)	30.8 ± 12.7	(5.3–50.9)	0.65
deMv-angle-final (°)	34.3 ± 14.9	(11.1–60.3)	40.8 ± 13.4	(13.6–66.4)	0.20
VD-baseline (%)	54.3 ± 7.0	(42.4–63.0)	54.8 ± 5.5	(39.2–61.2)	> 0.99
VD-final (%)	55.9 ± 7.1	(47.5–67.4)	53.0 ± 6.8	(39.2–59.8)	0.24
NFLD angle-baseline (°)	17.4 ± 14.3	(5.2–52.7)	20.7 ± 13.3	(0–45.1)	0.51
NFLD angle-final (°)	18.9 ± 15.3	(5.9–54.9)	23.9 ± 11.6	(7.0–48.7)	0.30

Data are presented as mean ± standard deviation (SD) with the minimum and maximum values in parentheses.

*CMvD* peripapillary choroidal microvasculature dropout, *deMv* decreased superficial retinal microvasculature, *DH* disc hemorrhage, *IOP* intraocular pressure, *NFLD* nerve fiber layer defect, *OCTA* optical coherence tomography angiography, *VD* vessel density.

*Analyzed using linear mixed-effect modeling between NFLD without DH and NFLD with DH.

**Figure 2:**
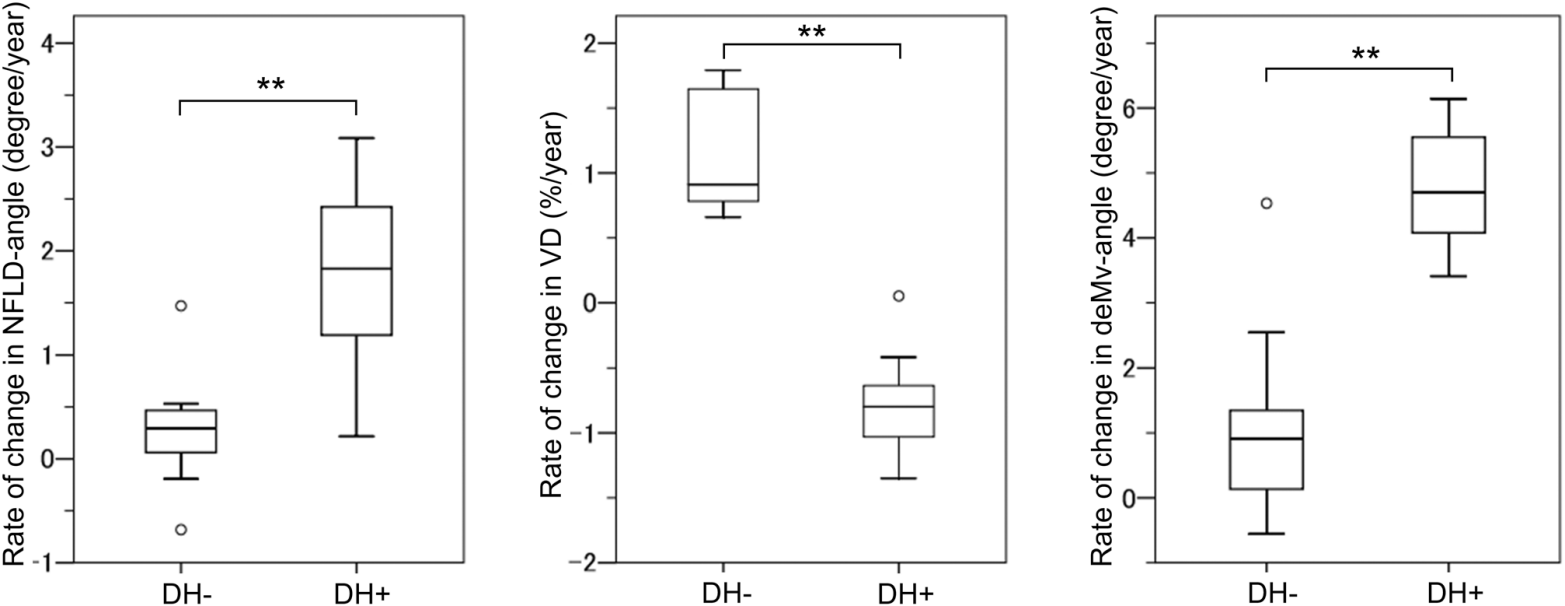
Box plots illustrating the distribution of the rates of changes in NFLD-angle (degree/year), VD (%/year), and deMv-angle (degree/year). Rates of changes in NFLD-angle and deMv-angle are adjusted by IOP, and the rate of VD change is adjusted by IOP and SSI. The medians are represented by horizontal lines in the white boxes. Boxes represent the interquartile range between the first and third quartiles. **P-value < 0.001.

Change rates of NFLD-angle, VD, and deMv-angle were evaluated using multivariable mixed-effects models including the history of DH and mean IOP (Table 3). Signal strength index (SSI) was also included as a variable in VD assessments. The models showed that the presence of DH history was associated with an additional average NFLD-angle widening of 2.19 degree/year (95% confidence interval [CI], 0.33–4.06; *P* = 0.030), VD decrease of 1.88%/year (95% CI, 0.40–3.35; *P* = 0.015), and deMv-angle widening of 3.78 degree/year (95% CI, 2.01–5.55; *P* < 0.001). Figure 2 shows the distribution of the change rates in NFLD-angle, VD, and deMv-angle after adjusting for IOP. The rates of NFLD-angle widening, VD decrease, and deMv-angle widening were significantly larger in the DH group than the non-DH group (NFLD-angle, 1.79 ± 0.75 degree/year vs. 0.27 ± 0.47 degree/year, *P* < 0.001; VD, − 0.82 ± 0.34%/year vs. 1.15 ± 0.43%/year, *P* < 0.001; deMv-angle, 4.78 ± 0.84 degree/year vs. 1.08 ± 1.25 degree/year, *P* < 0.001). The rates of NFLD-angle widening, VD decrease, and deMv-angle widening were not significantly different between the superotemporal and inferotemporal quadrants (all *P* > 0.05) (Supplementary Table 2).

**Table 3:** Results of multivariable mixed effects model analysis for longitudinal changes in NFLD-angle, VD, and deMv-angle in NFLD quadrants.

	Rate of NFLD-angle change		Rate of VD change		Rate of deMv-angle change	
	Coefficients (95% CI)	*P* value	Coefficients (95% CI)	*P* value	Coefficients (95% CI)	*P* value
Time, year	− 1.63 (− 7.31, 4.05)	0.58	− 3.69 (− 13.44, 6.06)	0.46	1.01 (− 4.29, 6.32)	0.37
DH history, yes	2.20 (− 8.03, 12.43)	0.68	− 0.33 (− 4.88, 4.22)	0.89	− 0.44 (− 9.47, 8.58)	0.92
DH history × time	2.19 (0.33, 4.06)	0.030	− 1.88 (− 3.35, − 0.40)	0.015	3.78 (2.01, 5.55)	< 0.001
Mean IOP, per mmHg	0.09 (− 1.87, 2.05)	0.93	0.24 (− 0.79, 1.27)	0.65	0.17 (− 1.58, 1.93)	0.85
Mean IOP × time	0.13 (− 0.27, 0.54)	0.54	− 0.10 (− 0.43, 0.23)	0.56	− 0.00 (− 0.38, 0.38)	0.99
SSI, per unit	n/a		0.11 (− 0.02, 0.24)	0.094	n/a	
SSI × time	n/a		0.08 (− 0.04, 0.21)	0.20	n/a	
Intercept	16.88 (− 11.40, 45.17)	0.25	43.40 (26.75, 60.05)	< 0.001	29.99 (4.76, 55.22)	0.030

Rates of changes in deMv-angle and NFLD-angle are adjusted by IOP, and the rate of VD change is adjusted by IOP and SSI.

*CI* confidence interval, *deMv* decreased superficial retinal microvasculature, *DH* disc hemorrhage, *IOP* intraocular pressure, *NFLD* nerve fiber layer defect, *SSI* signal strength index, *VD* vessel density, *n/a* not applicable.

**Figure 3:**
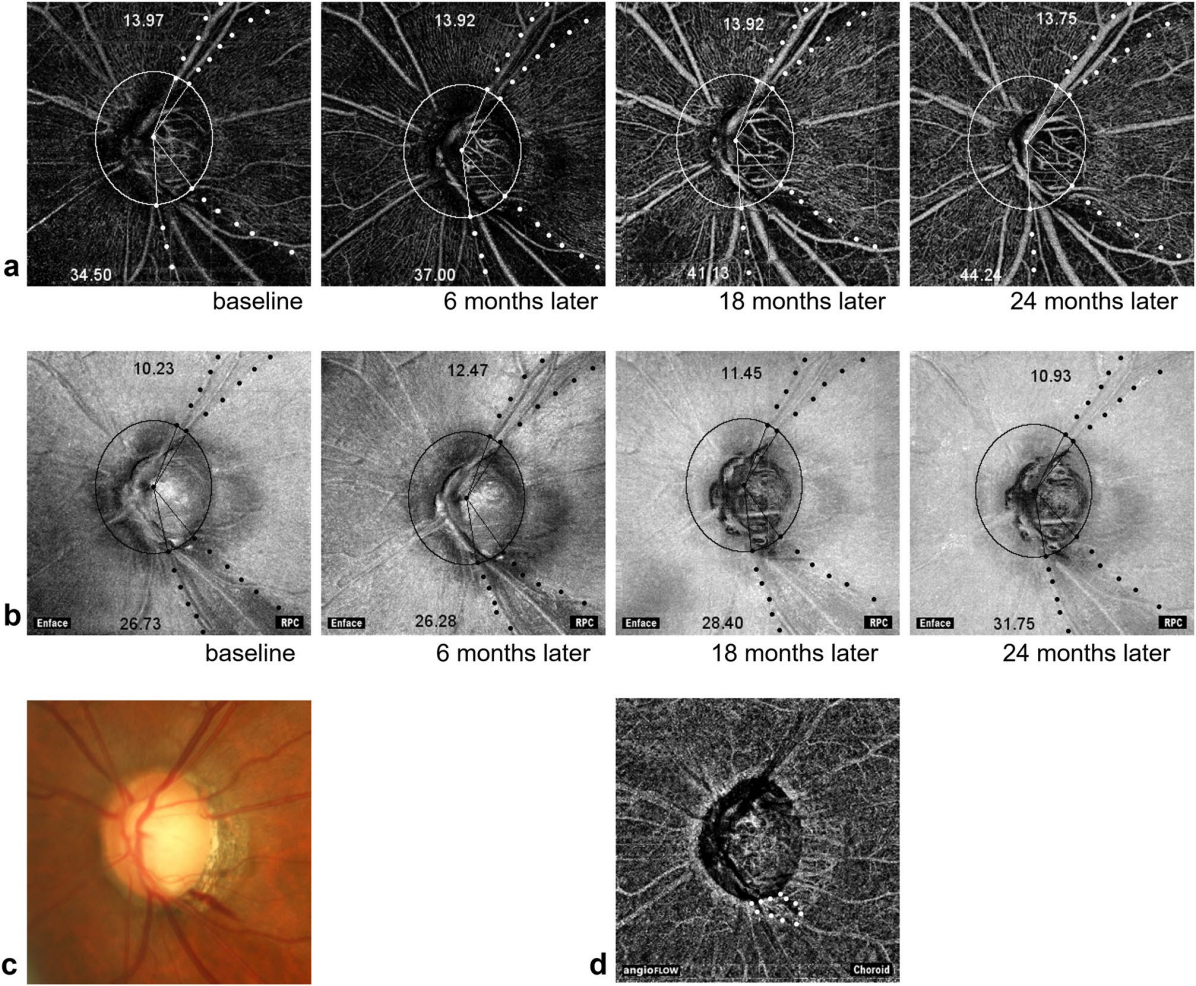
Representative images on longitudinal OCTA examination of a glaucomatous eye. (**a**) OCTA images obtained at the inner retinal layer show the longitudinal changes in deMv-angle of a 73-year-old man. Inferotemporal NFLD had 13 episodes of DH. Superotemporal NFLD had no DH episode. The numbers in the figure represent the angles of deMv in each NFLD. The deMv-angle of the inferotemporal NFLD changes more than the superotemporal one. (**b**) OCT en face images show the longitudinal changes in NFLD of the same eye. The numbers in the figure represent the angles of NFLD. The angle of inferotemporal NFLD changes more than the superotemporal one. RPC = Radial peripapillary capillary. (**c**) Disc photograph at baseline shows disc hemorrhage in inferotemporal NFLD. (**d**) Choroidal OCTA image of the same eye showing choroidal microvascular dropout (white dotted line).

Figure 3 shows a representative case with inferotemporal NFLD with DH history and superotemporal NFLD without DH history. Longitudinal widenings of NFLD-angle and deMv-angle were observed in the inferotemporal NFLD, but were not apparent in the superotemporal NFLD.

**Figure 4:**
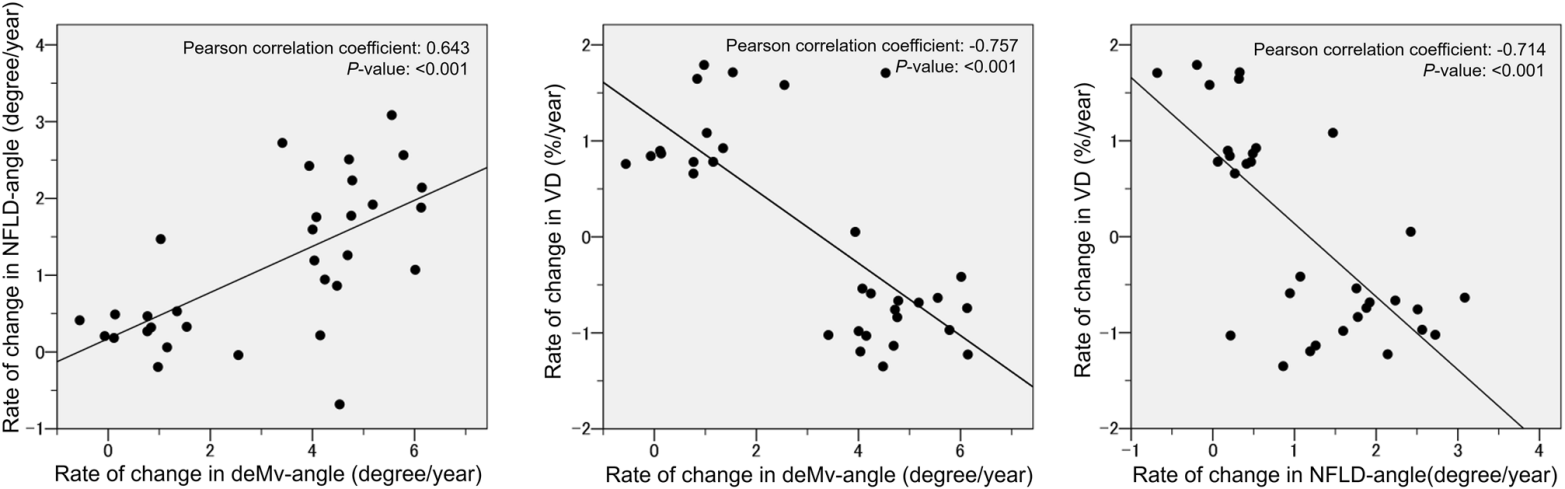
Scatterplots illustrating the linear associations between the rates of changes in NFLD-angle, VD, and deMv-angle. Rates of changes in NFLD-angle and deMv-angle are adjusted by IOP, and the rate of VD change is adjusted by IOP and SSI. These 3 parameters show significant linear correlations (NFLD-angle and deMv-angle, r = 0.643, *P* < 0.001; VD and deMv-angle, r = − 0.757, *P* < 0.001; NFLD-angle and VD, r = − 0.714, *P* < 0.001).

Figure 4 shows the scatterplots of the linear associations among the change rates of NFLD-angle, VD, and deMv-angle. These three parameters showed significant linear correlations (NFLD-angle and deMv-angle, r = 0.643, *P* < 0.001; VD and deMv-angle, r = − 0.757, *P* < 0.001; NFLD-angle and VD, r = − 0.714, *P* < 0.001).

## Discussion

The current study showed that a history of DH was a significant factor for site-specific progression of blood flow impairment in NFLD. The decrease in VD and widening of deMv-angle were significantly correlated with the widening of NFLD, suggesting that assessments of microvasculature using OCTA could be helpful to detect glaucoma progression.

Previous studies have shown the association between glaucoma progressions according to function ^9,11,12,17^ and structure^13,14,18^. The location of DH also is known to relate spatially to the progressive localized thinning of the RNFL^13,14^, widening of the NFLD^19,20^, and focal visual field progression^9,11^. Recent advancements in OCTA have enabled assessments of the microvasculature. There have been some reports that VD measured using OCTA can be useful to assess glaucoma progression^21^. Recently, Nitta et al.^22^ showed that a decrease in peripapillary VD was significantly associated with DH occurrence in patients with normal tension glaucoma, which is consistent with our results. However, the association between microvasculature reduction and RNFL thinning has not been fully clarified. Longitudinal assessments may be necessary to elucidate this issue.

We used the deMv-angle to investigate the longitudinal decrease in superficial retinal microvasculature. This parameter was first described by Lee et al.^8^ using the term “vascular impairment.” They showed that “vascular impairment” (deMv) was almost identical to NFLD in primary open angle glaucoma (POAG) eyes having a localized RNFL defect. In contrast, we found that the deMv was 14.5 degrees larger on average than the NFLD measurements in the current study (deMv-angle, 35.74 ± 13.73; NFLD-angle, 21.23 ± 13.69; *P* < 0.001). The reason for this discrepancy is not clear. One possible reason is the difference in the method used for the measurement of NFLD. NFLD was evaluated using red-free fundus photographs in the previous report, whereas OCT en face images were used in the current study. Another reason might be the difference in the threshold level between these methods. In any case, the change rate of the widening of deMv-angle was significantly associated with that of the widening of NFLD, which indicated that superficial microvasculature reduction was highly correlated with structural damage in NFLD.

In the current study, the change rates of NFLD-angle, VD, and deMv-angle had significant correlations with each other, which suggested that both OCTA and OCT parameters could be useful to detect glaucoma progression. Both OCTA and OCT could be valid and feasible assessments for glaucoma progression, although it was not clarified in the current study. Shoji et al.^21^ reported that a significant decrease was more detectable in macula VD than in ganglion cell complex thickness in some glaucomatous eyes. Moghimi et al.^23^ showed the possibility that in OCTA, VD was less affected by floor effects, and no further structural change could be detected in OCT-based thickness measurements. This evidence indicates the possibility of the different utility of these methods. Further studies should be conducted to clarify this issue.

This study has several limitations. First, the number of eyes analyzable was small. Nonetheless, change rates of all examined parameters (NFLD-angle, VD, and deMv-angle) were significantly different between the two groups, and their close relationships were detected with this small sample size. Second, because the line between the normal retina and the area of deMv to detect deMv-angle were manually evaluated, the values for deMv-angle could be different between graders. However, because the ICCs for these parameters were excellent, we believe that the results can be acceptable.

In conclusion, significant changes in NFLD-angle, VD, and deMv-angle were detected in NFLD with DH history more than in NFLD without DH history, and change rates of these parameters were significantly correlated with each other. This suggests that OCTA and OCT measurements can be used to detect glaucoma progression. Further studies are needed to determine the relationship between vascular and structural measurements and the usefulness of OCTA measurements in clinical practice.

## Methods

This longitudinal observational study was conducted at the Glaucoma Clinic of the Kyoto University Hospital. The study adhered to the tenets of the Declaration of Helsinki and was approved by the Institutional Review Board and Ethics Committee of the Kyoto University Graduate School of Medicine. Written informed consent was obtained from all the patients.

### Participants

The participants in this study consisted of primary open angle glaucoma (POAG) and preperimetric glaucoma (PPG) patients with DH history in either eye, who had OCTA examination at Glaucoma Clinic of the Kyoto University Hospital between March 1, 2015, and August 31, 2017. Inclusion criteria of this study were: (1) open angles on gonioscopy and best-corrected visual acuity of 20/40 or better at baseline to ensure high imaging quality. (2) Underwent more than three high-quality examinations of OCT and OCTA (signal strength index: SSI > 50). (3) The presence of NFLD confirmed using red-free image of fundus photo at the first OCTA examination. (4) Followed up with fundus photograph at least 3-month interval to detect the occurrence of DH. NFLD without DH in subjects met the above inclusion criteria were included as a control group. Exclusion criteria of this study were: (1) eyes with coexisting uveitis, retinal disease, or non-glaucomatous optic neuropathy. (2) eyes with any history of intraocular surgery including cataract surgery and glaucoma surgery.

The patients had undergone a comprehensive ophthalmic examination including measurement of best-corrected visual acuity (using a 5-m Landolt chart), slit-lamp examination, measurement of axial length (IOLMaster 500, Carl Zeiss Meditec, Dublin, CA), central corneal thickness (SP-3000, Tomay, Tokyo, Japan), Goldmann applanation tonometry, gonioscopy, indirect ophthalmoscopy, dilated slit-lamp examination of the optic nerve head, fundus photography, stereo disc photography (using a 3-Dx simultaneous stereo disc camera, Nidek, Gamagori, Japan), standard automated perimetry (Humphrey Visual Field Analyzer, Carl Zeiss Meditec) with the 24-2 Swedish Interactive Threshold Algorithm standard program^6^.

### Optic coherence tomography angiography

The optic nerve and peripapillary area were imaged using a commercially available OCTA device (AngioVue; OptoVue, Fremont, CA, USA). Each image covered an area of 4.5 × 4.5 mm and 3.0 × 3.0 mm centered on the optic disc. Each B-Scan contained 216 A- scan. To produce images of perfused vessels, the Split Spectrum Amplitude Decorrelation Angiography software algorithm was employed^7,24^. The OCTA images were coregistered with OCT B-scans that were obtained concurrently to enable visualization of both the vasculature and structure in tandem. The area of deMv was assessed by determining the presence of a region of decreased vasculature in the inner retina using the en face angiogram. Using the internal limiting membrane as a plane of reference, a slab with a uniform thickness that included the RNFL, ganglion cell layer, and inner plexiform layer was manually determined from the entire OCTA data sets using the coregistered OCT B-scans in each eye^8^.

The peripapillary region was defined as a 500-μm-wide elliptical annulus extending from the optic disc boundary, and segmentation of the peripapillary area was performed using the intrinsic software provided by OptoVue. Vessel density was defined by the percentage area occupied by vessels, measured using the intensity-based thresholding feature of the software, which adopted the same method of calculation as that previously reported^24^.

### Measurement methods of NFLD-angle, VD, and deMV-angle

In reference to previous reports^8,19^, NFLD-angle was measured by identifying the two points at which the borders of an NFLD area met the clinical optic disc margin (Fig. 1a), using 3.0 × 3.0 enface image of OCT. Lines were then drawn that connected the disc center and the two points, and the angular distance between these two lines was defined as NFLD-angle (Fig. 1b).

In the same way, deMv-angle was measured by identifying the two points at which the borders of the deMv area met the clinical optic disc margin and the disc center, using 3.0 × 3.0 enface image of OCTA (Fig. 1c).

As for VD, the optic disc was divided into six areas (blue dotted lines) using the software. In this study, we measured the VD of the superotemporal or inferotemporal areas (white dotted lines) in the location of NFLD (Fig. 1d).

### Statistical analysis

Values were presented as mean ± SD for continuous variables. Variables were compared using linear-mixed effects modeling, where eyes were nested within subjects to properly adjust for eyes from the same individual exhibiting similar measurements. The significance of differences between the groups was determined after Bonferroni correction.

Linear mixed-effects modeling was used to evaluate the rates of changes in NFLD-angle (degree/year), VD (%/year), and deMv-angle (degree/year). Details of the use of these models for assessment of longitudinal changes in glaucoma have been previously described^13,14,25–27^. In brief, models were first fit with objective parameter measurements (NFLD-angle, VD, or deMv-angle) as a response variable, whereas time, group (the presence of DH history), and a time-group interaction were defined as fixed effects. The variable, time, was measured as time in years from the first OCT and OCTA examination. Random intercepts and slopes were used to account for repeated measurements over time, where eyes (right or left) and locations (superotemporal or inferotemporal) were nested within subjects to properly adjust for NFLDs from the same individual exhibiting similar measurements. Then, multivariable models were evaluated with mean IOP throughout the follow-up period and SSI (in case of VD), which potentially affect rates of change in objective parameters, to evaluate relationships between objective parameter measurements over time and the presence of DH history. Two-way interactions between time and mean IOP and SSI (for VD) were used to determine whether these were significantly associated with change in objective parameter measurements over time. IOP-adjusted rates of changes in NFLD-angle and deMv-angle, and IOP-SSI-adjusted rates of VD change, were used for analyses. The statistical analyses were performed using the R package ‘lme4′ with R version 3.3.1 (http://www.r-project.org) and SPSS Version 24 software (IBM Corp., Armonk, New York, USA. *P* values less than 0.05 were considered statistically significant.

### Interobserver reproducibility of NFLD-angle and deMv-angle

To evaluate the interobserver reproducibility of NFLD-angle and deMv-angle, all NFLDs included in the current study were evaluated independently by two examiners (YO and EN) blinded to any information other than OCT enface images or OCTA, and intraclass correlation coefficients [ICCs (2,1)] were calculated.

## Supplementary Information

Supplementary Table 1.

Supplementary Table 2.

Supplementary Information.

## Data Availability

The raw data of the study are available at http://www.nature.com/srep.
