# Supplementary material for: Longitudinal changes in superficial microvasculature in glaucomatous retinal nerve fiber layer defects after disc hemorrhage
Source: Sci Rep. 2020 Dec 16;10:22058. doi: 10.1038/s41598-020-79151-y (PMC7744505; doi:10.1038/s41598-020-79151-y)
Supplement: Supplementary file 1 — Supplementary Table 1. [file 41598_2020_79151_MOESM1_ESM.docx]

**Longitudinal Changes in Superficial Microvasculature in Glaucomatous Retinal Nerve Fiber Layer Defects after Disc Hemorrhage**

Yoko Okamoto, Tadamichi Akagi, Kenji Suda, Takanori Kameda, Masahiro Miyake, Hanako Ohashi Ikeda, Eri Nakano, Akihito Uji, Akitaka Tsujikawa

**Supplementary Table 1. Comparisons of NFLD-angle, VD, and deMv-angle at Baseline between in Superotemporal and Inferotemporal Quadrants.**

|  | Superotemporal quadrant | Inferotemporal quadrant | *P* value* |
| --- | --- | --- | --- |
| Baseline NFLD-angle (Total), degree | 17.0±15.0 (0-52.7) | 20.8±12.7 (0- 45.1) | 0.44 |
| Baseline NFLD-angle (non-DH group), degree | 18.7±16.7 (5.1-29.0) | 16.2±12.5 (5.2-22.5) | 0.75 |
| Baseline NFLD-angle (DH group), degree | 15.0±14.1 (0-38.4) | 23.6±12.5 (0-45.1) | 0.21 |
| *P* value** (baseline NFLD-angle) | 0.68 | 0.054 |  |
| Baseline VD (Total), % | 54.8±4.9 (45.7-60.6) | 54.5±6.9 (39.2- 63.0) | 0.96 |
| Baseline VD (non-DH group), % | 54.5±5.6 (45.7-60.6) | 54.2±8.6 (42.4-63.0) | 0.94 |
| Baseline VD (DH group), % | 55.1±4.6 (49.3-60.0) | 54.6±6.1 (39.2-61.1) | 0.97 |
| *P* value** (baseline VD) | 0.19 | 0.97 |  |
| Baseline deMv-angle (Total), degree | 29.8±12.2 (12.7-54.6) | 33.0±12.8 (5.3-50.9) | 0.48 |
| Baseline deMv-angle (non-DH group), degree | 30.5±15.0 (12.7-54.6) | 35.2±10.2 (21.9-46.6) | 0.51 |
| Baseline deMv-angle (DH group), degree | 28.9±9.2 (16.0-35.9) | 31.8±14.4 (5.3-50.9) | 0.58 |
| *P* value** (baseline deMv-angle) | 0.081 | 0.59 |  |

Data are presented as mean ± standard deviation with the minimum and maximum values in parentheses.

deMv = decreased superficial retinal microvasculature ; DH = disc hemorrhage; NFLD = nerve fiber layer defect; VD = vessel density.

* analyzed using linear mixed-effect modeling between superotemporal and inferotemporal quadrants; ** analyzed using linear mixed-effect modeling between NFLD with DH and NFLD without DH.
