# Supplementary material for: Longitudinal changes in superficial microvasculature in glaucomatous retinal nerve fiber layer defects after disc hemorrhage
Source: Sci Rep. 2020 Dec 16;10:22058. doi: 10.1038/s41598-020-79151-y (PMC7744505; doi:10.1038/s41598-020-79151-y)
Supplement: Supplementary file 2 — Supplementary Table 2. [file 41598_2020_79151_MOESM2_ESM.docx]

**Longitudinal Changes in Superficial Microvasculature in Glaucomatous Retinal Nerve Fiber Layer Defects after Disc Hemorrhage**

Yoko Okamoto, Tadamichi Akagi, Kenji Suda, Takanori Kameda, Masahiro Miyake, Hanako Ohashi Ikeda, Eri Nakano, Akihito Uji, Akitaka Tsujikawa

**Supplementary Table 2. Comparisons of Change Rates of NFLD-angle, VD, and deMv-angle between Superotemporal and Inferotemporal Quadrants.**

|  | Superotemporal quadrant | Inferotemporal quadrant | *P* value* |
| --- | --- | --- | --- |
| Change rate of NFLD-angle (Total), degree/yr | 1.05 ± 0.96 (0.46, 1.63) | 1.18 ± 1.04 (0.67, 1.68) | 0.52 |
| Change rate of NFLD-angle (non-DH group), degree/yr | 0.37 ± 0.54 (-0.13, 0.86) | 0.18 ± 0.41 (-0.20, 0.56) | 0.62 |
| Change rate of NFLD-angle (DH group), degree/yr | 1.84 ± 0.69 (1.11, 2.57) | 1.76 ± 0.81 (1.24, 2.27) | 0.79 |
| *P* value** (baseline NFLD-angle) | 0.004 | <0.001 |  |
| Change rate of VD (Total), %/yr | 0.18 ± 1.06 (-0.45, 0.82) | 0.37 ± 0.54 (-0.13, 0.86) | 0.37 |
| Change rate of VD (non-DH group), %/yr | 1.08 ± 0.44 (0.67, 1.49) | 1.21 ± 0.44 (0.79, 1.62) | 0.34 |
| Change rate of VD (DH group), %/yr | -0.86 ± 0.23 (-1.10, -0.62) | -0.80 ± 0.39 (-1.05, -0.55) | 0.63 |
| *P* value** (baseline VD) | 0.014 | <0.001 |  |
| Change rate of deMv-angle (Total), degree/yr | 2.55 ± 2.12 (1.27, 3.83) | 3.57 ± 2.08 (2.57, 4.58) | 0.15 |
| Change rate of deMv-angle (non-DH group), degree/yr | 0.81 ± 0.95 (-0.08, 1.69) | 1.35 ± 1.53 (-0.06, 2.76) | 0.43 |
| Change rate of deMv-angle (DH group), degree/yr | 4.59 ± 0.67 (3.89, 5.30) | 4.88 ± 0.92 (4.28, 5.46) | 0.054 |
| *P* value** (baseline deMv-angle) | <0.001 | <0.001 |  |

Data are presented as mean ± standard deviation with the 95% confidence interval in parentheses.

deMv = decreased superficial retinal microvasculature ; DH = disc hemorrhage; NFLD = nerve fiber layer defect ; VD = vessel density.

* analyzed using linear mixed-effect modeling between superotemporal and inferotemporal quadrants; ** analyzed using linear mixed-effect modeling NFLD with DH and NFLD without DH.
